# Effects of Climate Change on Soil Organic Matter C and H Isotope Composition in a Mediterranean Savannah (*Dehesa*): An Assessment Using Py-CSIA

**DOI:** 10.1021/acs.est.3c01816

**Published:** 2023-09-08

**Authors:** Layla M. San-Emeterio, Lorena M. Zavala, Nicasio T. Jiménez-Morillo, Ignacio M. Pérez-Ramos, José A. González-Pérez

**Affiliations:** † Instituto de Recursos Naturales y Agrobiología de Sevilla, Consejo Superior de Investigaciones Científicas (IRNAS-CSIC), Av. Reina Mercedes 10, 41012 Sevilla, Spain; ‡ Universidad de Sevilla, MED Soil Res. Group, Dpt. Cristalografía, Mineralogía y Química Agrícola, Facultad de Química, C/Prof Garcia Gonzalez 1, 41012 Sevilla, Spain; § University of Évora, Instituto Mediterrâneo para a Agricultura, Ambiente e Desenvolvimento (MED), Núcleo da Mitra, Ap. 94, 7006-554 Évora, Portugal

**Keywords:** δ^13^C, δ^2^H, analytical pyrolysis, Mediterranean soil, biomarkers, climate change

## Abstract

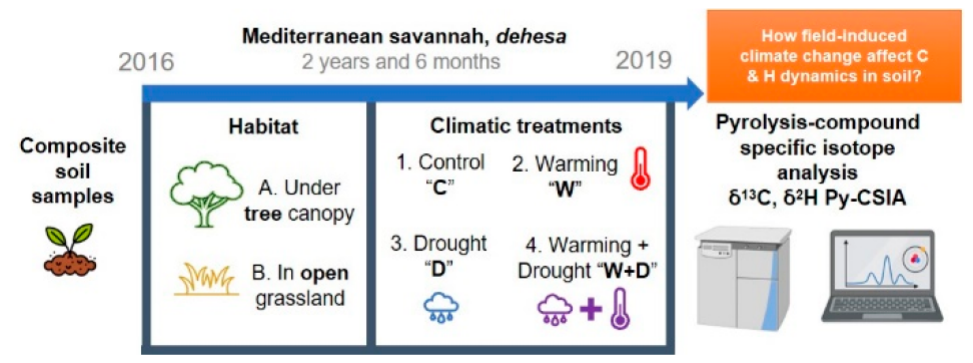

*Dehesas* are Mediterranean agro-sylvo-pastoral systems sensitive to climate change. Extreme climate conditions forecasted for Mediterranean areas may change soil C turnover, which is of relevance for soil biogeochemistry modeling. The effect of climate change on soil organic matter (SOM) is investigated in a field experiment mimicking environmental conditions of global change scenarios (soil temperature increase, +2–3 °C, W; rainfall exclusion, 30%, D; a combination of both, W*+*D). Pyrolysis-compound-specific isotope analysis (Py-CSIA) is used for C and H isotope characterization of SOM compounds and to forecast trends exerted by the induced climate shift. After 2.5 years, significant δ^13^C and δ^2^H isotopic enrichments were detected. Observed short- and mid-chain *n*-alkane δ^13^C shifts point to an increased microbial SOM reworking in the W treatment; a ^2^H enrichment of up to 40‰ of lignin methoxyphenols was found when combining W+D treatments under the tree canopy, probably related to H fractionation due to increased soil water evapotranspiration. Our findings indicate that the effect of the tree canopy drives SOM dynamics in *dehesas* and that, in the short term, foreseen climate change scenarios will exert changes in the SOM dynamics comprising the biogeochemical C and H cycles.

## Abstract 2

Pyrolysis-compound-specific stable isotope analysis (Py-CSIA) was useful for forecasting SOM evolution in Mediterranean “*dehesas*” under global climate change scenarios.

## 1 Introduction

Soil organic matter (SOM) constitutes the largest pool of terrestrial organic carbon (C), accounting for ca. 2/3.^1^ Though the overall balance of the carbon cycle is close to equilibrium, soils may act as a C source or sink depending on land use or environmental factors. The combination of this large C pool with a relatively fast dynamic has made research on SOM of prime environmental interest.^2^

A major source of uncertainty when estimating future effects on climate comes from the effect of warming on SOC.^3,4^ Soil respiration increase with increasing temperature points to acceleration of SOC decomposition,^5^ decrease of SOC storage and release of CO_2_ as a positive feedback to global warming,^6,7^ and the magnitude and duration of this effect is uncertain. On the other side, drought is believed to decrease SOC by reducing plant biomass and rhizodeposition.^8−10^ Also, drought may exert SOC losses by microbial mineralization through a reduction of microbial activity and decreased litter quality.^11^ However, recurrent or severe wetting and drying cycles can start biological activity pulse and CO_2_ release “priming effects” that in turn may exert net SOC losses under severe droughts.^12−14^ Therefore, understanding how soil responds to global change is relevant because several drivers may be acting simultaneously, and SOC responses might not be a simple additive function. Progress in understanding the potential effects of these global change factors on SOC stocks can be achieved with the use of C isotope techniques, increased cross-site observational studies, and experimental manipulation that integrate multiple global change factors and modeling.^3^

Mediterranean areas represent the most vulnerable ecosystems to climate change due to their ecotone condition and marked seasonality. Therefore, this study is focused in the study of soils from Mediterranean savannas, widely known in Spain as *dehesas*; typical agro-sylvo-pastoral systems, characterized by the scattered presence of oak trees (*Quercus ilex*, *Q. suber*) with a continuous herbaceous cover.^15^ This ecosystem covers 3 million ha in Spain and ca. 11% of the global land surface.^16^*Dehesas* are characterized by diverse microclimatic areas, where the presence of trees alter the local environmental conditions believed to buffer extreme temperatures, diminish evaporation, and increase soil carbon content.^17^ The tree canopy also indirectly alters the ecosystem functioning, where herbaceous species usually develop morphological and physiological traits that allow them to tolerate the shade and competition caused by trees.^18^ As a result, SOM molecular structure and dynamics can differ in the two main habitats of the *dehesa*: in open grassland and under the tree canopy.^19^ This diversity of microhabitats also accounts for relevant soil environmental drivers such as soil temperature and water content and its availability to plants.^20^

The study of SOM light elements’ stable isotopes can provide information about biomass sources and the prevailing environmental conditions when they were biosynthesized and about processes involved in SOM formation and evolution. The stable carbon isotopic signature (δ^13^C) of terrestrial plants largely depends on their photosynthetic pathway to discriminate ^13^CO_2_ (C_4_ vs C_3_ plants; angiosperms vs gymnosperms),^21,22^ but also reflects smaller differences in plant physiology such as stomatal conductance and water use efficiency under different environmental conditions.^23^ In soils, enriched ^13^C normally indicates more degraded SOC.^24,25^ On the other hand, the δ^2^H primarily reflects the properties of the water that the plants absorbed from soil.^26^ This δ^2^H in soil water depends on climatic conditions (temperature, evaporation, and precipitation) and varies depending on the global and local hydrological cycle, causing plant biomolecules bearing δ^2^H values in the range of the precipitation water.^27^

It is possible nowadays to determine stable isotope values for specific biogenic compounds and specific biomarkers, e.g. *n*-alkanes, fatty acids, lignin components, or polysaccharides, that provide the opportunity to better understand soil carbon dynamics,^28^ whose values may also be affected by biosynthesis pathways^29,30^ and, after deposition in soil, by processes of evolution and microbial activity.^24^

Although it is known that some compound such as waxes and long-chain alkanes are isotopically unchangeable during biodegradation,^31^ other studies have reported that different biomarkers can exhibit distinct patterns of carbon isotopic fractionation during biodegradation experiments.^32,33^ On the other hand, H has various molecular forms in soil, determining its organic or inorganic form and how it can be exchanged with ambient water vapor;^34,35^ H covalently bound to C in organic molecules is considered stable and nonexchangeable.^36^ Among these, nonexchangeable H of *n*-alkanes and lignin methoxyphenols can enlighten processes related to paleoclimatic reconstruction,^37^ SOM degradation processes,^38^ and source vegetation and origin.^21,39^

Uncertainties in the turnover of SOM compounds hinder the implementation of innovative analytical techniques for elucidating this topic. There are several wide fingerprinting tools that could be applied in SOM characterization, such as conventional analytical pyrolysis gas chromatography/mass spectrometry (Py-GC/MS)^19,40^ and isotope ratio mass spectrometry (IRMS).^41−43^ However, conventional bulk isotopic values are not sufficient to assess SOM changes under the influence of diverse processes.^44^ Compound-specific isotope analysis (CSIA) is also used^45,46^ but usually implies the use of extraction and derivatization steps that will result in analytical complications and the addition of extra carbon and hydrogen to the specific compounds, affecting their isotope signature.^47^ A relatively new variant of the CSIA technique includes pyrolysis (pyrolysis-compound-specific isotope analysis, Py-CSIA) and allows the direct analysis of soil samples with no need for extraction or derivatization.^48^ In the field of biogeochemistry, this technique has demonstrated a wide potential when applied to complex samples.^49−53^ These studies also demonstrated that pyrolysis products represented the isotope composition of their precursors and that the technique did not produce isotopic fractionation as previously noted.^54−56^

In this work, the effects of temperature and drought on the isotopic composition of major elements (C and H) in specific SOM biomarker groups are studied. Soil samples from both representative habitats in this ecosystem, in open grassland and under the tree canopy, combined with climatic variations were studied. The working hypothesis is that forecasted environmental shifts due to climate change will be reflected in SOM stable isotope composition (C and H). This, in turn, will provide opportunities to identify environmental drivers of SOM dynamics and to better understand the effect of future and more severe climate events in Mediterranean savannah and elsewhere. To the best of our knowledge, this is an innovative approach where a direct compound-specific isotope analysis is applied for the first time in extracting environmental information encompassed within SOM molecular diversity.

## 2 Materials and Methods

### 2.1 Site Description

The study area was an evergreen oak *dehesa* in Pozoblanco, Córdoba, southwestern Spain (38°20′47.8″N 4°48′57.0″W) at 675 m a.s.l. The climate is dry Mediterranean-type with warm dry summers and humid cold winters. Mean annual temperature and rainfall are 15.7 °C and 439 mm yr^–1^, most occurring from October to May (IFAPA meteorological station, Hinojosa del Duque; data from the 2017–2021 period). The soil is classified as Eutric Cambisol,^57^ characterized by a sandy-loamy texture and the absence of carbonates. The vegetation is dominated by sclerophyllous evergreen oak (*Quercus ilex* L.) with a tree density of 14.5 ± 1.3 trees ha^–1^ and an herbaceous understory of annual native pasture dominated by *Hordeum murinum* (L.), *Senecio vulgaris* (L.), *Bromus madritensis* (L.), and *Sinapis alba* (L.).^18^

### 2.2 Experimental Design and Soil Sampling

The experimental design was set in October 2016, consisting of a full factorial field experiment where the main factors were habitat type and climatic treatments. The two most representative habitat types in the savanna ecosystems were chosen: under the canopy of *Quercus ilex* (hereafter *tree*) and nearby open grasslands (hereafter *open*). Four permanent plots of 4 × 6 m were installed in each habitat type and fenced to exclude livestock access. In each habitat, four climate scenarios (treatments) were simulated: (1) *control* (C), without any manipulation; (2) *warming* (W), using methacrylate open top hexagonal chambers (0.65 m^2^) to force an increase of 2–3 °C according to climate forecasting models^58^ (SRES A-2 model by the IPCC, 2022); (3) *drought* (D), using rainfall-exclusion shelters with six “V”-shaped methacrylate chanaletts (0.14 m wide; 20° inclination) intercepting 30% of the precipitation based on the same IPCC scenario; (4) a combined *warming and drought* (W+D) scenario, to assess the impact of both climatic stressors.^59,60^

The effects of the treatments in soil temperature and moisture have been studied in detail.^18^ In summary, both soil temperature and moisture were lower under the tree canopy than in open grassland and these differences were more pronounced in spring. The treatments W and W+D significantly increased soil temperature by about 2 °C, and soil moisture was significantly higher in C and W than in D and W+D in spring, but only in the open.

Soil sampling was conducted in spring (late April) 2019, three growing seasons after the experimental trial was set up, taking the 10 uppermost centimeters of soil using an auger. In total, 8 experimental units (2 habitat types × 4 climatic treatments) were sampled and studied, where three samples were taken per experimental unit and combined in a composite sample. The resulting samples were air-dried, sieved to fine earth (2 mm), and stored at room temperature. Prior to analysis, all samples were ground to a fine powder and homogenized using an agate mortar.

### 2.3 Elemental (C) and Isotopic (δ^13^C, δ^2^H) Bulk Analysis

For δ^13^C and δ^2^H bulk isotopic determination, 0.5 mg of soil samples was enveloped into tin and silver capsules, respectively. The samples were analyzed in triplicate (*n* = 3) using an IRMS EA IsoLink System (Thermo Fisher Scientific, Bremen, Germany), consisting of a EA coupled, via a ConFlo IV Interface unit, to a Delta V Advantage isotope ratio mass spectrometer (IRMS). For C isotope analysis, the tin cups were flush-combusted and flush-reduced concurrently under a He carrier steam and oxygen pulse at 1020 °C in a quartz combustion reactor filled with chromium oxide (Cr_2_O_3_), silvered cobaltous/cobaltic oxide (Ag-Co_3_O_4_) and reduced copper (Cu). For H isotopes, the samples were analyzed in the IRMS pyrolysis reactor, which consists of an outer ceramic (Al_2_O_3_) tube and an inner glassy-carbon reactor tube filled with high-purity glassy-carbon granulates and silver and quartz wool. The silver cups were dropped under a steam of He into the pyrolysis reactor tube held at 1450 °C. Evolved gases from both analyses were passed through a 10 cm long glass column filled with a mixture of anhydrous magnesium perchlorate (Mg(ClO_4_)_2_) in order to dry the gas in both analyses and Carbosorb to trap any CO_2_ generated only during the pyrolysis reaction. Gases were directed through a stainless-steel gas chromatography column (3 m in length and 4 mm in diameter) packed with a Porapak stationary phase at 70 °C for the separation of CO_2_ and H_2_, whose isotopic composition was analyzed in the IRMS. Pure CO_2_ and H_2_ gas were inserted into the He carrier flow as pulses of the reference gas (250 mL min^–1^) for each determination.

The stable isotope abundances are reported in the delta (δ) notation (δ^13^C, δ^2^H) in variations relative to an international measurement standard. The isotope value is defined according to the equation^61^
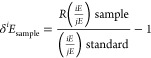
where *R* is the molar ratio of the heavy *iE* to light, and *jE* the most abundant isotope of the chemical element *E* (^13^C/^12^C for δ^13^C values; ^2^H/^3^H for δ^2^H values). The δ values are reported in units per mil (‰). Isotopic values were corrected using the appropriate standards recognized by the International Atomic Energy Agency (IAEA). The analytical precision and accuracy of bulk δ^13^C and δ^2^H were typically less than ±0.5 and 1.5‰, respectively.

The total C content was determined by the dry combustion method using the same instrument specified above (EA IsoLink) in CN analyzer mode by quantifying the evolved gases from the combustion in a thermal conductivity detector (TCD).

For hydrogen measurements, both bulk and CSIA, the H^3+^ factor determined daily during the measurement period was within the range 4.76–5.17. Lastly, for both bulk and CSIA δ^2^H determinations, soil samples were oven-dried (80 °C) to remove water and stored in a desiccator until analysis.

### 2.4 Pyrolysis-Compound-Specific Isotopic Analysis (δ^13^C, δ^2^H Py-CSIA)

The δ^13^C and δ^2^H of individual compounds was determined by direct pyrolysis-compound-specific isotopic analysis (Py-CSIA). The samples (15 mg) were pyrolyzed using a double-shot pyrolyzer Model 3030D; Frontier Laboratories Ltd., Fukushima, Japan) attached to a GC Ultra instrument fitted with a IsoLink IRMS System (Thermo Fisher Scientific, Bremen, Germany) with two microreactors, one for thermal conversion (TC) set at 1420 °C, and another for combustion (C) set at 1020 °C. The system was coupled to a Delta V Advantage IRMS via a ConFlo IV universal interface (Thermo Scientific, Bremen, Germany). The chromatographic separation of the pyrolysis compounds was performed using an Agilent J&W HP-5 ms UI capillary column (30 m × 250 μm × 0.25 μm). The GC oven temperature was held at 50 °C for 1 min and then increased to 100 °C at 20 °C min^–1^, increased from 100 to 300 °C at 10 °C min^–1^, and maintained at 300 °C for 10 min. The carrier gas used was He at a controlled flow of 1 mL min^–1^. Each chromatographic compound was gasified in the IsoLink System, and pure CO_2_ and H_2_ were mixed into the He carrier flow as pulses of reference gases. As a daily routine, the isotopic values were calibrated against a saturated *n*-alkane mixture using the reference substances A_7_ and C_4_ (Biogeochemical Laboratories, Indiana University, USA) for δ^13^C and δ^2^H measurements, respectively.^62^ The linear correlation between standard and measured (IRMS) from the *n*-alkane mixtures was used to derive sample isotopic values relative to the VSMOW and VPDB scales for the δ^2^H and δ^13^C values. Standard and measured isotopic values fitted well along a straight line with a linear regression *R*^2^ of no lower than 0.99 in all cases. The internal precision of δ^13^C and δ^2^H was ±0.7 and 4‰, respectively. Background subtractions and isotope abundances were calculated by using ISODAT 3.0 software (Thermo Scientific, Bremen, Germany).

The identification of specific chromatographic compounds was done by comparing and matching the mass spectra obtained with the Py-CSIA analysis with Py-GC/MS chromatograms obtained using the same pyrolysis and chromatographic conditions.^63^ A detailed semiquantitative Py-GC/MS assessment can be found in San-Emeterio et al.^19^

### 2.5 Statistical Analysis

An analysis of variance (two-way factorial ANOVA) was performed when the assumptions of normal distribution and homogeneity of variance were met using the Kolmogorov–Smirnov test and the Levene test, respectively. This analysis was applied to analyze the individual and combined effects of habitat type and climatic treatments as independent variables and bulk and individual isotope values as dependent variables. Significant differences were identified by Tukey’s HSD tests and denoted with letter coding, with different letters representing significant differences between groups. Partial ETA squared values (“ETA”, hereafter) are also given as a measure to show the effect size of independent variables for group mean differences. When data did not meet the normality or homogeneity requirements, even after log transformation, a nonparametric Scheirer–Ray–Hare test was used for treatment comparisons and their interactions along with a Dunn posthoc test for multiple nonparametric comparisons. A Wilcoxon signed rank test for paired comparisons was used for evaluating differences between isotopic composition of *n*-alkanes retrieved from biomass debris and bulk SOM. These statistical analyses were made with a 95% confidence level using SPSS 20.0 (SPSS Inc., Chicago, USA) and RStudio (version 2022.02.3). The “rcompanion”^64^ and “FSA”^65^ packages were used for the Scheirer–Ray–Hare test.

## 3 Results and Discussion

### 3.1 Elemental (C) and Isotopic (δ^13^C and δ^2^H) Analysis

The isotopic composition of SOM in Mediterranean pastures was strongly conditioned by the effect of habitat. Table 1 shows the results of the elemental analysis of soil C content (%) and bulk stable isotope composition of bulk soil C and H. For C, significant differences were found between habitats, with enriched δ^13^C values (0.7‰ higher) and higher C content (1.5% higher) under the tree canopy. This could be explained by biomass inputs coming from the tree (δ^13^C_biomass_ = −27.4 ± 0.43‰), together with heterotrophic SOM degradation and reworking that usually produces a δ^13^C enrichment.^66,67^ These differences associated with the habitat were also found for δ^2^H bulk values, with significantly enriched values (3.6‰ higher) under the tree canopy. This probably points to a better water availability in the open grassland soil (more depleted δ^2^H values) due to less evapotranspiration in leaves and soil.^68,69^ However, no differences were detected due to the climatic treatments for any of the bulk isotope parameters. Effects of the climate treatments were noticeable only under the tree canopy, with significantly higher C content in “D” plots (3.5 ± 0.2 vs 2.6 ± 0.2 as average from the rest of treatments), whereas no differences were found among climatic treatments in the open habitat. This increase compared to open grassland may be attributed to the accumulation of a less evolved SOM in the former, resembling fresh biomass, as previously found for *dehesa* SOM under trees.^19^

**Table 1:** Carbon Content and C and H Stable Isotope Composition of Bulk Soil Samples Collected in Two Different Habitat Types (under Tree and Open Grassland) and Four Experimental Climatic Treatments (Mean *n* = 3 ± Standard Error)^a^

	open			tree		
	C content (%)	δ^13^C (‰ V-PDB)	δ^2^H (‰ V-MOW)	C content (%)	δ^13^C (‰ V-PDB)	δ^2^H (‰ V-MOW)
C	1.1 ± 0.1^B^	–28.1 ± 0.3^B^	–84.6 ± 1.0^B^	2.4 ± 0.4^Aa^	–27.5 ± 0.2^A^	–81.8 ± 0.6^A^
W	1.2 ± 0.1^B^	–28.2 ± 0.4^B^	–84.9 ± 0.8^B^	2.4 ± 0.1^Aa^	–27.8 ± 0.1^A^	–81.7 ± 1.1^A^
D	1.3 ± 0.1^B^	–27.4 ± 0.5^A^	–84.5 ± 0.9^B^	3.5 ± 0.2^Ab^	–27.6 ± 0.2^A^	–79.4 ± 1.0^A^
W+D	1.5 ± 0.2^B^	–28.2 ± 0.3^B^	–83.5 ± 1.3^B^	2.7 ± 0.2^Aa^	–27.5 ± 0.1^A^	–80.5 ± 1.1^A^

a Abbreviations: C, control; W, warming; D, drought; W+D, warming + drought. Different letters indicate significant differences (Two-way ANOVA; *p* < 0.05): uppercase letters indicate differences between habitats; lowercase letters indicate differences between climatic treatments within a same habitat.

### 3.2 Pyrolysis-Compound-Specific Isotope Analysis (δ^13^C and δ^2^H Py-CSIA)

**Figure 1:**
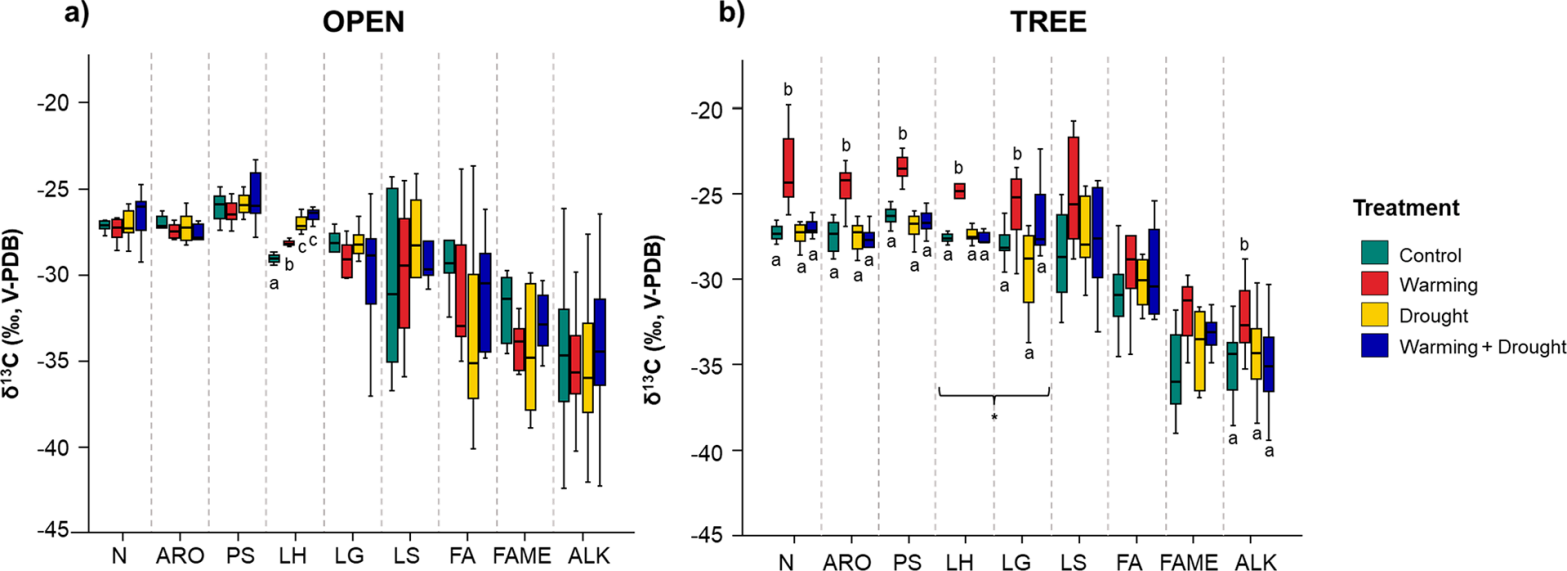
Distribution of δ^13^C Py-CSIA values (average expressed as ‰; *n* = 3) of (a) open and (b) tree habitat, for the different biogenic compounds identified by pyrolysis. Abbreviations: N, nitrogen compounds; ARO, aromatics from unknown origin; PS, polysaccharides; LH, *p*-hydroxyphenyl lignin units; LG, guayacil lignin units; LS, syringyl lignin units; FA, fatty acids; FAME, fatty acid methyl ester; ALK, *n*-alkanes. Error bars indicate standard errors. Treatments with the same letters indicate no significant differences between different composting times for the same biogenic group. Asterisks indicate significant differences between habitats.

It was possible to assign the δ^13^C composition for a total of 58 major pyrolysis compounds, which varied from −19.3 to −49.0‰ (Table S1). The identified pyrolysis compounds were biogenically derived from polysaccharides (furans, cyclopentanediones, anhydrosugars), lignin (methoxyphenols from guaiacyl (LG), syringyl (LS), and *p*-hydroxyphenyl (LH) units), and aliphatic compounds such as alkane/alkane doublets (ALK), fatty acids (FA) and fatty acid methyl esters (FAME). Other minor compounds were also present, such as nitrogen-containing compounds (N) (indole and diketodipyrrole) and other aromatic compounds (ARO) (phenols and benzenes) (Figure 1). The most ^13^C-depleted compounds were the aliphatic compounds (ALK, FA, and FAME) (−35.2 ± 0.6‰), whereas PS showed a heavier C isotope composition (−25.4 ± 0.4‰); lignin methoxyphenols (LG, LS, and LH) had intermediate values (−28.2 ± 0.8‰) (Figure 1). These differences among biogenic groups are mainly caused by fractionation during distinct plant metabolic paths, i.e., plant polysaccharides hold a heavier ^13^C-enriched carbon in position 6 of the sugar molecules^70^ and lignin is usually ^13^C depleted compared with bulk SOM^53,71^ and these isotope signal are preserved after pyrolysis.^48^ Nonetheless, isotopic differences may also reflect the origin of the compound, microbial- or animal-derived compounds,^45^ i.e., short- to mid-chain alkanes, polysaccharides, or nitrogen-containing compounds, are usually also ^13^C enriched.

Significant differences between habitats were found only for the lignin guayacil units (LG), with more enriched δ^13^C (up to 2.5‰) values under the tree canopy. This ^13^C enrichment may be mainly attributed to the SOM source, which agrees with differences driven by the presence of the *Quercus* tree canopy, and hence distinct proportion of vegetation input, as well as additions from relatively ^13^C-enriched root tissue that may be also favoring additional fractionation by microbial decomposers.^72^ In this habitat type, significant differences among climatic treatments were also found, with more enriched values under W treatment for N, ARO, PS, and LG, compared to others (Figure 1b). This ^13^C enrichment supports the increase in soil temperature as driving the environmental factor controlling soil microbial activity. In fact, it has been reported that more advanced stages of microbial degradation of SOM driven by soil warming lead to an enrichment in ^13^C.^73−75^ Furthermore, it is observed that the ^13^C enrichment is precisely associated with smaller compounds, mainly short-chain alkyl compounds (Table 2), that may have a microbial origin. On the other hand, shifts toward ^13^C enrichment in compounds from plant origin such as lignin can only be explained by changes in the floristic composition of the plot or to an environmentally mediated plant stomatal conductance reduction.^76^ This may be the case for the observed trend of compound LH in the open prairie, for which significant differences were found for the three treatments.

**Table 2:** Carbon Isotope Compositions (Mean *n* = 3 ± Standard Error) of *n*-Alkanes/Alkenes Identified in SOM by Py-CSIA (‰, Relative to V-PDB)^a^

	open				tree			
	C	W	D	W+D	C	W	D	W+D
*n*-Alkenes								
C_19:1_	–26.3 ± 0.1	–25.4 ± 0.2	–27.7 ± 0.0	–27.1 ± 0.5	–32.6 ± 1.1^a^	–27.4 ± 0.8^b^	–27.1 ± 0.2^b^	–27.4 ± 1.1^b^
C_20:1_*	–36.5 ± 0.1	–32.2 ± 0.3	–30.4 ± 0.3	–29.2 ± 0.6	–34.2 ± 0.1	–33.0 ± 0.2	–36.3 ± 0.7	–35.1 ± 0.1
C_21:1_	–32.0 ± 0.3	–32.1 ± 0.3	–37.8 ± 0.6	–30.3 ± 0.4	–33.5 ± 0.0	–31.3 ± 0.6	–34.1 ± 1.1	–35.1 ± 0.2
C22:1	–31.8 ± 0.3	–31.0 ± 0.5	–32.8 ± 0.1	–33.8 ± 0.3	–33.1 ± 0.2	–30.5 ± 0.3	–33.1 ± 0.4	–32.9 ± 0.2
C23:1	–35.0 ± 0.9^a^	–30.1 ± 0.2^b^	–33.1 ± 0.3^a^	–35.6 ± 1.0^a^	–33.0 ± 0.8	–31.0 ± 0.7	–34.0 ± 0.1	–34.6 ± 0.9
C24:1	–34.7 ± 0.8^a^	–35.3 ± 0.5^a^	–31.2 ± 1.2^b^	–30.7 ± 2.5^b^	–33.0 ± 0.2	–32.5 ± 0.6	–33.1 ± 0.1	–33.4 ± 1.3
C25:1*	–38.6 ± 0.7^a^	–37.0 ± 0.3^b^	–35.7 ± 0.8^b^	–36.3 ± 0.1^b^	–34.1 ± 0.3	–32.8 ± 0.5	–33.7 ± 0.9	–34.8 ± 0.3
C26:1	–36.0 ± 0.5^a^	–32.9 ± 0.9^b^	–37.5 ± 0.4^a^	–35.0 ± 0.1^a^	–36.1 ± 0.1	–33.9 ± 0.3	–34.8 ± 0.2	–35.3 ± 0.1
*n*-Alkanes								
C_18_*	–36.4 ± 0.4	–36.3 ± 0.2	–37.5 ± 1.2	–34.9 ± 1.0	–36.6 ± 0.0^a^	–30.1 ± 0.4^b^	–27.3 ± 0.1^c^	–33.7 ± 1.6^b^
C_19_	–32.0 ± 0.6	–34.0 ± 0.5	–34.1 ± 0.1	–31.1 ± 0.4	–32.4 ± 0.4^a^	–28.1 ± 0.5^b^	–30.9 ± 0.3^a^	–35.5 ± 1.1^b^
C_20_	–34.6 ± 0.7	–34.0 ± 0.1	–32.2 ± 0.9	–32.8 ± 1.3	–38.3 ± 1.6^a^	–33.7 ± 0.1^b^	–35.0 ± 0.1^b^	–37.0 ± 0.5^a^
C_21_	–33.0 ± 0.4	–34.9 ± 0.0	–34.0 ± 1.0	–34.0 ± 0.4	–33.7 ± 0.3	–33.2 ± 0.8	–33.0 ± 0.7	–30.6 ± 0.3
C_22_	–28.2 ± 0.0	–31.2 ± 0.5	–30.5 ± 0.5	–31.2 ± 0.3	–34.3 ± 0.0^a^	–32.5 ± 2.8^a^	–30.9 ± 0.6^b^	–28.4 ± 0.2^c^
C_23_	–35.3 ± 0.2	–34.0 ± 2.0	–34.1 ± 0.1	–34.8 ± 1.6	–34.3 ± 0.0^a^	–27.9 ± 1.3^b^	–34.2 ± 0.7^a^	–33.7 ± 0.7^a^
C_24_*	–36.6 ± 0.4	–35.1 ± 0.6	–34.9 ± 0.2	–35.4 ± 0.0	–34.3 ± 0.2^a^	–30.6 ± 0.0^b^	–33.4 ± 0.1^a^	–34.8 ± 0.2^a^
C_25_*	–40.6 ± 1.2	–39.5 ± 0.8	–40.8 ± 0.2	–38.4 ± 0.7	–34.5 ± 0.1	–34.7 ± 0.5	–32.9 ± 0.6	–37.1 ± 0.6
C_26_*	–39.0 ± 0.8	–37.5 ± 0.9	–41.5 ± 0.7	–41.1 ± 1.3	–36.6 ± 0.7	–34.2 ± 0.4	–35.2 ± 0.3	–37.4 ± 0.2
C_27_	–38.8 ± 0.8^a^	–36.4 ± 0.1^b^	–38.4 ± 0.6^a^	–35.3 ± 1.8^b^	–38.8 ± 0.9^a^	–34.5 ± 0.3^b^	–34.7 ± 0.2^b^	–34.5 ± 0.1^b^
C_28_	–39.6 ± 0.3^a^	–37.0 ± 0.6^b^	–36.1 ± 0.7^b^	–37.3 ± 0.5^b^	–41.5 ± 0.5^a^	–35.6 ± 1.2^b^	–35.8 ± 0.2^b^	–37.1 ± 0.2^ab^
C_29_	–42.0 ± 0.5^a^	–36.2 ± 0.5^b^	–36.1 ± 0.3^b^	–35.9 ± 1.2^b^	–37.0 ± 0.3^a^	–34.4 ± 0.3^b^	–36.0 ± 0.4^a^	–35.1 ± 0.0^ab^
C_30_	–39.5 ± 0.3^a^	–31.9 ± 0.3^b^	–38.8 ± 0.9^a^	–28.0 ± 0.1^b^	–36.2 ± 0.1	–35.3 ± 0.1	–37.9 ± 0.2	–38.3 ± 0.4
C_31_	–43.2 ± 2.0^a^	–37.7 ± 0.4^b^	–36.3 ± 0.3^b^	–35.8 ± 2.3^b^	–36.9 ± 0.1^a^	–34.1 ± 0.1^b^	–37.5 ± 0.7^b^	–39.0 ± 0.2^b^

a Abbreviations: C, control; W, warming; D, drought; W+D, warming + drought. Lowercase letters indicate significant differences for the climatic scenarios within the same habitat; asterisks indicate significant differences according to the habitat (two-way ANOVA, *p* < 0.05).

**Figure 2:**
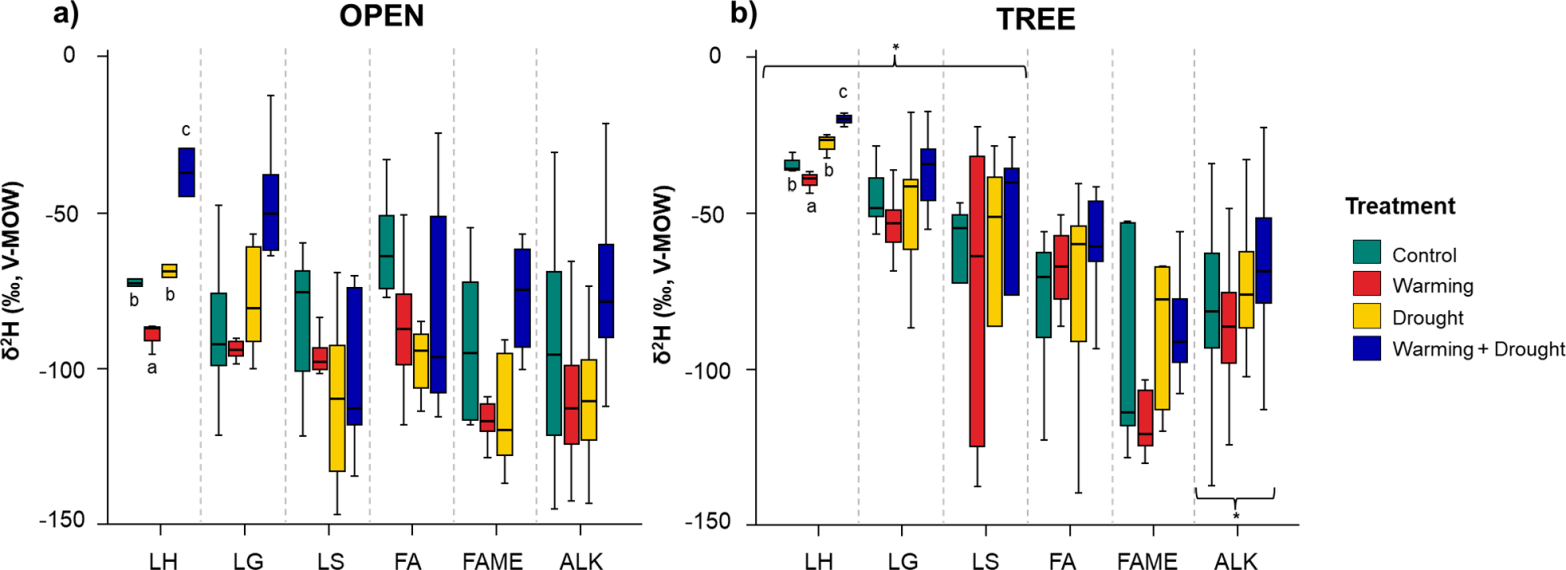
Distribution of δ^2^H Py-CSIA values (average expressed as ‰; *n* = 3) of (a) open and (b) tree habitat, for the different groups identified by pyrolysis, attributed to nonexchangeable biogenic groups. LH: *p*-hydroxyphenyl lignin units; LG: guayacil lignin units; LS: syringyl lignin units; FA: fatty acids; FAME: fatty acid methyl-ester; ALK: *n*-alkanes. Error bars indicate standard error. Treatments with the same letters indicate no significant differences between different composting times for the same biogenic group. “*” indicate significant differences between habitats. LS excluding δ^2^H values of propynylsyringol.

Despite Py-CSIA providing δ^2^H values for a total of 48 compounds (Table S2), only aliphatic and lignin compounds were considered for further analysis and discussion (Figure 2) due to their nonexchangeable H feature. Overall, all lignin units (LH, LG, LS) exclusively showed significant differences between habitats with more enriched values of δ^2^H under the tree canopy (up to 20 ‰, *p* = 0.000). As for ^13^C, The LH units were the only compounds that were notably influenced by climate manipulation, showing an enrichment in the “W+D” treatment. Neither δ^2^H values corresponding to FA and FAME were significantly influenced by any of the two factors. Lastly, δ^2^H of alkyl compounds varied significantly with the habitat, being more enriched under the tree canopy (Figure 2).

#### 3.2.1 Isotopic Composition (δ^13^C and δ^2^H) of Alkyl Compounds

Carbon and hydrogen isotopic compositions of the alkyl compounds are summarized in Tables 2 and 3, respectively. The δ^13^C composition of alkyl compounds were composed of C_18_–C_31_, including the unsaturated forms from C_19:1_ to C_26:1_. Generally, the δ^13^C values of long-chain (>C_24_) *n*-alkyl molecules ranged from −28.0 to −43.2 in open grasslands and from −30.6 to −41.5 under the tree canopy, which is consistent with the general ^13^C distribution of aliphatic compounds from C3 angiosperm trees.^21^ Two-way ANOVA showed no significant differences and little effect due to the habitat (*p* = 0.155, ETA 0.104) or climatic treatments (*p* = 0.113, ETA 0.074). However, by evaluating these differences according to each individual aliphatic compounds, a ^13^C enrichment under the tree canopy up to 5.0‰ was observed in some alkyl compounds (C_18_, C_20:1_, C_24_, C_25:1_, C_25_, and C_26_), mostly caused by the W treatment (Table 2).

**Table 3:** Hydrogen Isotopic Compositions (Mean *n* = 3 ± Standard Error) of *n*-Alkanes from Bulk Soil Identified by Py-CSIA (‰, Relative to V-MOW)^a^

	open				tree			
	C	W	D	W+D	C	W	D	W+D
C_19_	–73.0 ± 1.9	–105.5 ± 5.0	–102.0 ± 10.7	–62.2 ± 8.3	–101.0 ± 0.3^a^	–57.9 ± 0.6^b^	–53.8 ± 4.4^b^	–50.4 ± 1.1^b^
C_20_	–105.3 ± 5.0^a^	–89.6 ± 4.2^a^	–54.6 ± 5.1^b^	–31.0 ± 5.1^b^	–69.8 ± 3.9^a^	–79.7 ± 4.9a̲	–48.0 ± 4.5^b^	–31.3 ± 7.1^b^
C_21_*	–100.0 ± 1.7^a^	–120.2 ± 3.1^a^	–60.3 ± 3.7^b^	–54.7 ± 1.4^b^	–53.7 ± 3.6^a^	–38.7 ± 2.9^b^	–37.7 ± 3.2^b^	–29.4 ± 3.4^c^
C_22_	–120.7 ± 1.5	–96.1 ± 6.2	–125.7 ± 1.6	–102.5 ± 9.6	–147.2 ± 1.4^a^	–91.7 ± 5.2^b^	–87.4 ± 0.8^b^	–83.0 ± 11.0^b^
C_23_*	–122.2 ± 11.7	–143.3 ± 5.5	–167.8 ± 5.1	–148.0 ± 3.8	–137.0 ± 1.0^a^	–117.6 ± 2.0^a^	–91.1 ± 6.7^b^	–133.1 ± 0.8^a^
C_24_*	–127.9 ± 5.8^a^	–107.6 ± 1.8^a^	–125.3 ± 8.4^a^	–69.5 ± 5.1^b^	–87.9 ± 2.0^a^	–69.9 ± 7.6^b^	–91.9 ± 2.0^a^	–80.3 ± 1.1^b^
C_25_	–143.4 ± 2.9^a^	–107.5 ± 2.2^b^	–107.4 ± 1.0^b^	–37.5 ± 2.5^c^	–83.2 ± 1.4	–93.5 ± 3.6	–75.8 ± 2.9	–62.5 ± 2.0
C_26_	–150.1 ± 8.0^a^	–117.7 ± 3.9^a^	–92.5 ± 1.9^b^	–37.3 ± 1.2^c^	–83.8 ± 0.9^a^	–82.9 ± 1.9^a^	–37.1 ± 0.4^c^	–54.2 ± 4.7^b^
C_27_*	–136.2 ± 8.0^a^	–126.6 ± 9.5^a^	–82.7 ± 4.7^b^	–103.9 ± 12.7^a^	–92.7 ± 4.5^a^	–65.8 ± 1.2^b^	–68.1 ± 5.1^b^	–57.1 ± 1.0^b^
C_28_	–112.6 ± 2.1	–74.5 ± 10.0	–68.1 ± 10.7	–79.6 ± 0.8	–86.4 ± 1.5a̲	–61.9 ± 0.0^b^	–77.3 ± 4.0^b^	–76.1 ± 0.2^b^
C_29_	–113.7 ± 0.2^a^	–77.9 ± 7.9^b^	–76.4 ± 3.2^b^	–98.7 ± 2.5^a^	–87.4 ± 6.9^a^	–79.2 ± 1.9^a^	–76.6 ± 2.4^a^	–62.9 ± 4.7^b^
C_30_	–145.8 ± 3.6^a^	–114.6 ± 2.0^a^	–106.4 ± 0.5^a^	–63.5 ± 4.4^b^	–127.9 ± 2.1^a^	–103.9 ± 10.8^a^	–131.9 ± 0.1^a^	–67.6 ± 3.6^b^
C_31_*	–168.4 ± 5.5^a^	–92.4 ± 1.9^b^	–129.3 ± 0.9^a^	–81.7 ± 1.5^b^	–65.9 ± 3.0	–75.7 ± 1.1	–66.3 ± 2.0	–52.5 ± 1.7

a Abbreviations: C, control; W, warming; D, drought; W+D, warming + drought. Lowercase letters indicate significant differences for the climatic scenarios within the same habitat; asterisks indicate significant differences according to the habitat (Scheirer–Ray–Hare test, *p* < 0.05).

Interestingly, these climate-induced changes appeared mainly in open grasslands in regard to the unsaturated alkyl compounds (from C_23:1_ to C_26:1_), which exhibited an ^13^C enrichment in “W” treatment. The only exception was C_24:1_, with enriched values in plots subjected to drier conditions (in both D and W+D treatments). This contrasts with what is observed under the tree canopy, where only C_19:1_ showed a significant enrichment in those plots subjected to experimental climate manipulation compared to control.

For the saturated alkyl compounds, a higher number of *n*-alkanes showed significant responses to climate manipulation under the tree canopy compared with open habitat. The following compounds showed a ^13^C enrichment in response to a temperature increase (i.e., W and/or W+D plots): C_18–20_, C_22–24_, C_27–29_, and C_31_. On the other hand, long-chain alkanes from C_27_ to C_31_ exhibited greater δ^13^C values under climate manipulation, especially due to experimental warming. In addition, microbial activity during SOM reworking could also be responsible for the isotopic shifts of short- and mid-chain *n*-alkyl molecules in soils, as observed for C_18_, C_19_, C_23_, and C_24_ under tree canopy for W plots. Previous ^13^C incubation studies have proven that warming can substantially alter the stability of SOM.^3,75^ Hence, this suggests that long-chain *n*-alkyl molecules in soils may undergo specific indirect isotopic modification during accelerated microbial biodegradation associated with induced climate change (i.e., changes in the degradation of biomass inputs).^77^ In fact, microbial degradation of long-chain *n*-alkanes is not a rare phenomenon in natural settings due to heterotrophic reworking during degradation.^78,79^

Comparison between δ^13^C of long-chain *n*-alkanes (C_27_, C_29_, and C_31_) from biomass debris (Figure S1) and those identified from SOM was not significantly different under the two habitats or the climatic treatments. This supports the idea that the isotopic composition of the *n*-alkanes can be attributed to the contributions of biomass from each habitat. Therefore, the variation in δ^13^C found between the climatic treatments could be attributed to the direct or indirect effects of soil environmental shifts on the organic matter.^80^

Individual *n*-alkane δ^2^H compounds are presented in Table 3, encompassing C_19_–C_31_. The δ^2^H values of *n*-alkanes show a wide range from −29.4 to −168.4‰ and ^2^H-depletion relative to the precipitation values registered in that latitude, which is expected to be in the lighter part of the range given for the area (−41.7 to −23.4).^81^ Two-way ANOVA showed a statistically significant effect exerted by habitat (*p* = 0.000, ETA 0.203), with more enriched δ^2^H values under the tree canopy as well as for the climatic treatments (*p* = 0.000, ETA 0.125), with more enriched values (up to 10.3‰ as an average) in the W+D treatment. Partial ETA squared values showed that the relative impact of the habitat is shortly twice as strong as the climatic treatment.

**Figure 3:**
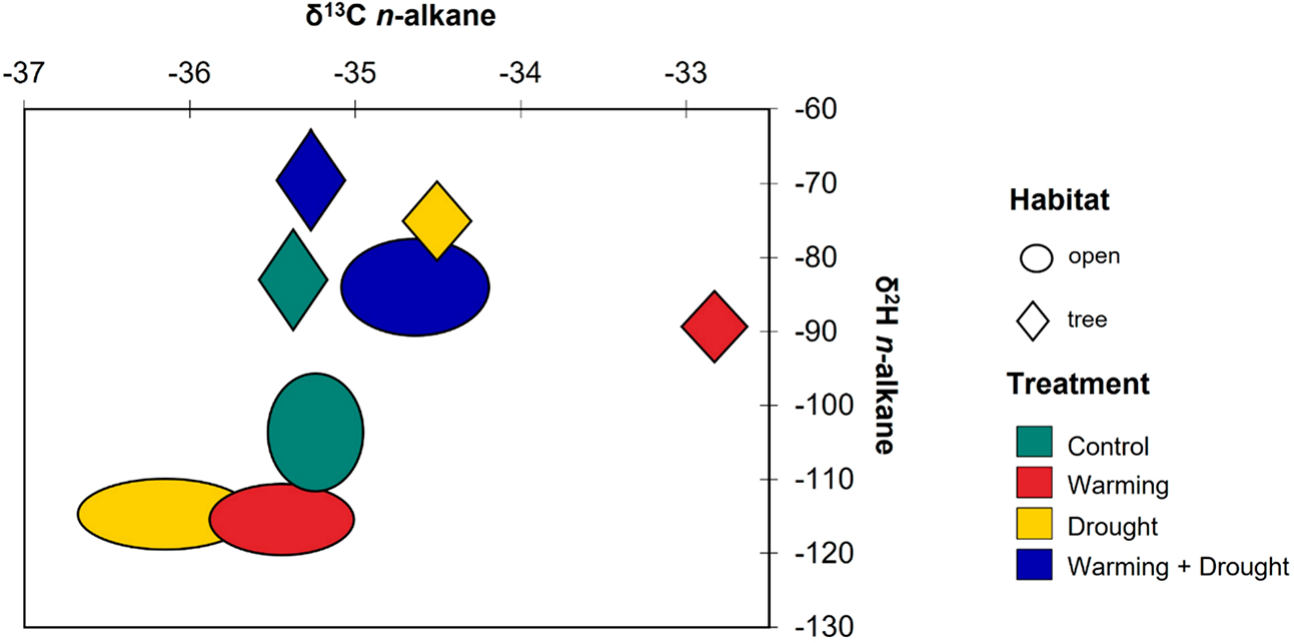
Distribution of compound-specific δ^2^H and δ^13^C *n*-alkanes data from soils under the two different factors. The size of the circles for the different samples denotes the standard error of isotopic values between individual *n*-alkanes and climatic treatments. The shape of the forms represents the two different habitats.

Comparing δ^2^H composition of individual *n*-alkanes, statistical differences for C_21_, C_23_, C_24_, C_27_, and C_31_ were found due to the habitat, again more enriched under the tree canopy (Table 3). Under the tree canopy, all compounds except for C_25_ and C_30_*n*-alkanes showed greater ^2^H enrichment in the W+D treatment. Lesser compounds showed significant differences in open grassland, though a remarkable number were also ^2^H-enriched, particularly again in W+D except for C_27_ and C_29_, which included the single D or W effects. A graphical relationship between compound-specific δ^2^H and δ^13^C *n*-alkane data is shown in Figure 3. It is observed that SOM from open pasture tended to have lighter δ^2^H values compared with those under tree canopy, except for the W+D treatment, which showed a significant ^2^H enrichment. Under the tree canopy, W and D showed significantly enriched δ^13^C values compared to C and W+D.

As we assume that there is no fractionation during water uptake from soil by roots,^82^ changes in δ^2^H values should be attributed to external, environmental (microhabitat and seasonality) factors.^83^ According to previous studies, possible factors that may control *n*-alkane δ^2^H values are microclimatic gradients such as relative humidity.^84^ As reported by field data (data not shown), relative humidity is higher under the tree canopy related to the open pasture, caused by differences in vegetation cover and exposure to solar radiation.^85^ In addition to the difference in soil humidity, differences in plant physiology may contribute to the observed isotopic variations. This isotopic difference has been interpreted because of the ecological differences of terrestrial plants, probably leading to different degrees of evapotranspiration, and thus a larger vapor pressure deficit between leaf stomatal apertures and the surrounding atmosphere.^86^ Grasses have to protect their growth zone from high evaporative demand during summer season (greater temperatures), whereas growing tree leaves escape a high evaporative demand by spring growth.^87^

#### 3.2.2 Isotopic Composition (δ^13^C and δ^2^H) of Lignin Methoxyphenols

**Figure 4:**
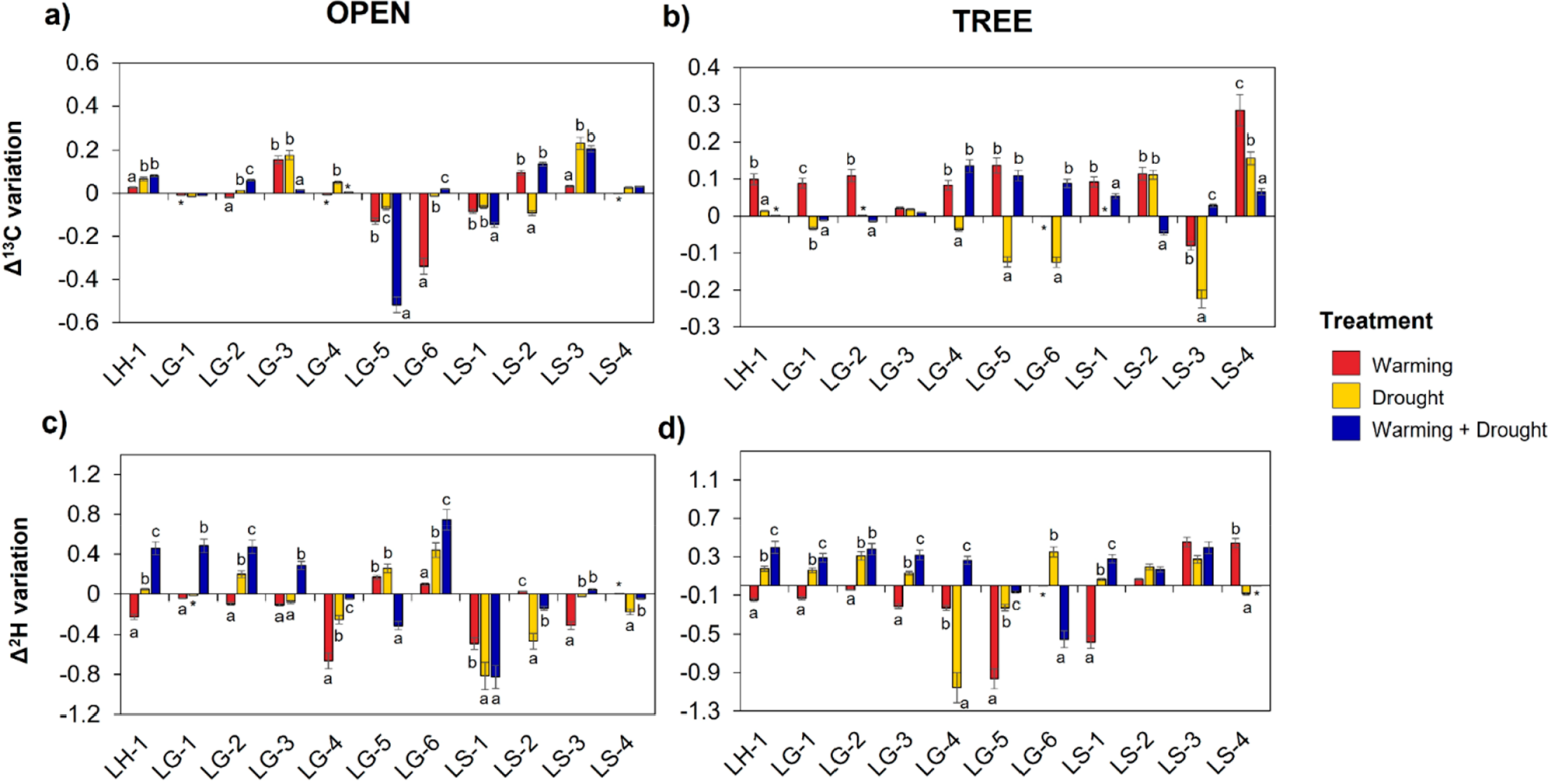
Changes in δ^13^C and δ^2^H isotope composition (expressed as Δ**)** relative to the control in three climatic treatments. Difference = (Treatment – Control)/Control. Data points represents means (*n* = 3 ± standard error). Letters represent significant differences (Scheirer–Ray–Hare Test, *p* < 0.05) in the same compounds between climatic treatments. Asterisks indicate values out of graphical representation. Abbreviations: LH-1, vinylphenol; LG-1, guaiacol; LG-2, methylguaiacol; LG-3, vinylguaiacol; LG-4, vanillin; LG-5, eugenol; LG-6, acetoguaiacone; LS-1, syringol: LS-2, propynylsyringol; LS-3, methoxyeugenol; LS-4, acetosyringone.

Relative changes in the δ^13^C and δ^2^H isotope composition of lignin methoxyphenols are depicted in Figure 4. As previously found, significant shifts for δ^13^C values were mainly caused by the habitat type, with more enriched values under the tree canopy (Scheirer–Ray–Hare Test, *p* = 0.03). In the open prairie, significant ^13^C depletion was found in plots subjected to W treatment for LG units with an allyl chain and acetyl group (i.e., eugenol and acetoguaiacone, respectively) (Figure 4a). A remarkable ^13^C depletion was also observed in eugenol in the W+D plots. In general, isotopic variation was less pronounced in the open prairie than under trees. On the other hand, warmer conditions caused a significant, more pronounced ^13^C enrichment for all methoxyphenols except for methoxyeugenol. This ^13^C enrichment was also observed for longer-chain LG units (vanillin, eugenol, and acetoguaiacone) but also in the W+D treatment (Figure 4b). Conversely, the D treatment induced a significant ^13^C depletion for all lignin units except for vanillin, eugenol, acetoguaiacone, and acetosyringone.

Similar isotopic shifts were also detected for δ^2^H values, with a general enrichment of δ^2^H under the tree canopy (Scheirer–Ray–Hare test, *p* = 0.02). Climate manipulation also altered the δ^2^H isotopic composition, with slight differences depending on the habitat type. In open grasslands, a conspicuous ^2^H enrichment was detected for all methoxyphenols (except for LS units; Figure 4c). This enrichment was mainly observed in the W+D treatment for all LH and LG units, which conversely caused a depletion for the LS units. This effect was also observed under the tree canopy (Figure 4d), where almost all lignin biomarkers showed an ^2^H enrichment in D and W+D; treatments. Nonetheless, W treatment depicted the opposite effects, with ^2^H-depleted values for most lignin methoxyphenols except for methoxyeugenol and acetosyringone.

The isotopic shifts in both ^13^C and ^2^H observed in lignin methoxyphenols are most likely a result of fractionations during biosynthesis.^88^ Since plant C isotope composition mainly depends on plant physiology status and the environmental conditions prevailing when growing, such ^13^C enrichment is probably due to a reduced stomatal conductance caused by warming to the grass communities under the tree canopy. For H isotope composition, differences are highly attributed to soil–water dynamics and reflect the δ^2^H values of the water taken by the plant.^89^ These δ^2^H enrichments might reflect soil water enriched in ^2^H due to higher evapotranspiration or, to a lesser extent, to plant physiological differences due to amplified leaf-water ^2^H enrichment prior to its biosynthetic incorporation into the plant.^90^

The possibility that the spatial variability in biomass inputs due to habitat might be reflected in lignin isotope variation cannot be fully excluded. However, preferential degradation of some monomers, as has been previously reported,^91,92^ and microbial-mediated molecular changes should also be addressed. Two feasible explanations are found for these isotopic differentiation of lignin units: (1) n the first place, these methoxyphenols are produced through a more direct pathway of incorporation in growing biomass;^93^ (2) their biodegradation rate as part of SOM led to an enrichment of residual substrates.^94^ Since preferential complex enzymatic reactions have taken place because of indirect changes in microbial dynamics, hence the lighter isotopes can be more easily utilized by microbes.^95^

Ultimately, biomarker data comprising alkyl and lignin SOM compounds suggest that climatic treatments effectively resulted in a change in ecosystem hydrology causing enriched δ^2^H values as found in the W+D treatment in the tree habitat. Despite the fact that it could be hypothesized that the drought effect could be buffered under the tree canopy, it has been proved that under the tree canopy, the combined effect of warming and drought is more intense compared to that in open grassland. There are two possible explanations for this trend: (1) the combination of warming and drought causing a higher evaporation at the interface between air and soil surface compared to other climatic treatments, leading to ^2^H enrichment of soil water, that is supported by the ^2^H values recorded for biomarkers up to 40 ‰ heavier, and (2) the lower H fractionation found in the open habitat could be due to distinct soil structure that in turn may be affecting water dynamics, supported by the fact that soils with high exposure to solar radiation cause changes in soil aggregate stabilization and formation,^96^ affecting the way water circulates within the soil–air interface.^97^

Overall, the direct analysis of δ^13^C and δ^2^H in specific SOM biomarkers through pyrolysis (Py-CSIA) combined with field experiments mimicking future forecasted environmental conditions allows us to assert that SOM dynamics in Mediterranean *dehesas* is prone to changes and will be affected in the short term under future climatic change scenarios. Also, this experimental approach facilitated unraveling processes of SOM dynamics under the new environmental conditions—that include increasing temperatures and more intense/frequent drought cycles—that are soon reflected in SOM chemistry and that will ultimately affect the C and H biogeochemical cycles in these agroecosystems.

Further analyses are encouraged to cover other aspects related to SOM evolution, as influenced by climate change. More specifically and since SOM dynamics and isotopic composition are driven largely by changes in soil microbial communities, microbiological analyses will be needed to complement the findings of this work: i.e. classical culture-dependent microbiology approaches or DNA-based analyses focused on next-generation sequencing (NGS) and its combination with stable isotopes of specific microbial-derived lipids (e.g., ^13^C PLFAs).

## Supporting Information Available

The following files are available free of charge. The Supporting Information is available free of charge at https://pubs.acs.org/doi/10.1021/acs.est.3c01816.

Detailed lists of the isotope composition of carbon and hydrogen for all identified SOM pyrolysis products and comparison of δ^13^C values for C_27_, C_29_, and C_31_*n*-alkane biomarkers retrieved Py-CSIA from bulk SOM and aboveground biomass (PDF)

## Abbreviations

SOM: soil organic matter
Py-GC/MS: pyrolysis coupled to gas chromatography–mass spectrometry
Py-CSIA: pyrolysis-compound-specific isotope analysis
IRMS: isotope ratio mass spectrometry
