# Supplementary material for: Effects of Climate Change on Soil Organic Matter C and H Isotope Composition in a Mediterranean Savannah (Dehesa): An Assessment Using Py-CSIA
Source: Environ Sci Technol. 2023 Sep 8;57(37):13851–62. doi: 10.1021/acs.est.3c01816 (PMC10515479; doi:10.1021/acs.est.3c01816)
Supplement: Supplementary file 1 — es3c01816_si_002.pdf [file es3c01816_si_002.pdf]

Effects of climate change on soil organic matter C and H isotope composition in a Mediterranean savannah (dehesa): An assessment using Py-CSIA..

*Layla M. San-Emeterio†§, Lorena M. Zavala§, Nicasio T. Jiménez-Morillo†‡, Ignacio M. Pérez-Ramos†, José A. González-Pérez†**

†Instituto de Recursos Naturales y Agrobiología de Sevilla, Consejo Superior de Investigaciones Científicas (IRNAS-CSIC), Av. Reina Mercedes 10, 41012 Sevilla, Spain

§Universidad de Sevilla, MED Soil Res. Group, Dpt. Cristalografía, Mineralogía y Química Agrícola, Facultad de Química, C/Prof Garcia Gonzalez 1, 41012 Sevilla, Spain

‡University of Évora, Instituto Mediterrâneo para a Agricultura, Ambiente e Desenvolvimento (MED), Núcleo da Mitra, Ap. 94, 7006-554 Évora, Portugal

Summary

Figure S1. Comparison of $\delta^{13}C$ values for a) C_{27}, b) C_{29} and c) C_{31} n-alkanes biomarkers retrieved Py-CSIA from bulk SOM and aboveground biomass. Statistical differences ($p < 0.05$) are denoted within each type of habitat. Error bars represent standard error (n = 4)..S3

Table S1. List of pyrolysis products, with their retention times (RT, in minutes), biogenic compound (origin) and their $\delta^{13}C$ values in each of the climatic treatment within each habitat (average expressed as ‰; $n = 3 \pm$ standard error)………………………………S4

Table S2. List of pyrolysis products, with their retention times (RT, in minutes), biogenic compound (origin) and their $\delta^{2}H$ values in each of the climatic treatment within each habitat (average expressed as ‰; $n = 3 \pm$ standard deviation)......................................S6

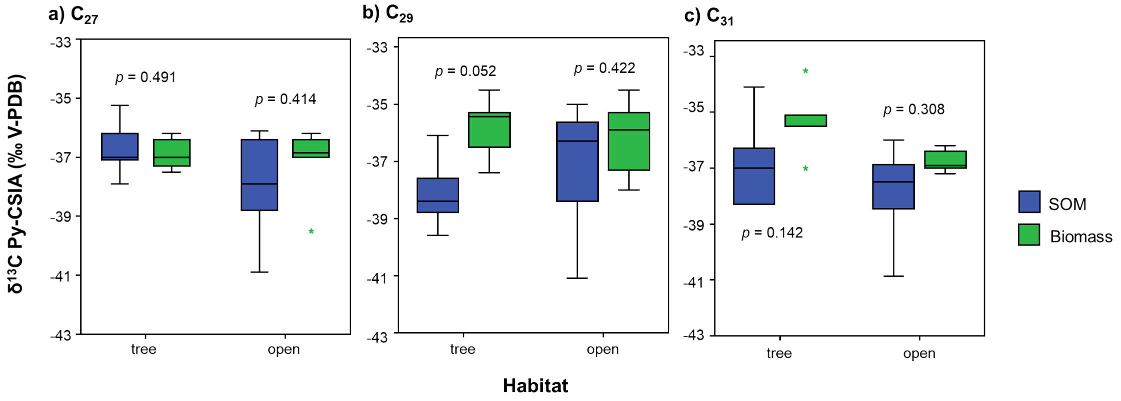

Figure S1. Comparison of $\delta^{13}C$ values for a) C_{27}, b) C_{29} and c) C_{31} *n*-alkanes biomarkers retrieved Py-CSIA from bulk SOM and aboveground biomass. Statistical differences ($p < 0.05$) are denoted within each type of habitat. Error bars represent standard error ($n = 4$).

Table S1. List of pyrolysis products, with their retention times (RT, in minutes), biogenic compound (origin) and their $\delta^{13}C$ values in each of the climatic treatment within each habitat (average expressed as ‰; *n* = 3 ± standard error).

			Open				Tree			
**RT**	**Compound**	**Origin**	**C**	**W**	**D**	**W+D**	**C**	**W**	**D**	**W+D**
2.45	Pyridine	N	-27.2±0.2	-26.9±0.2	-26.1±0.2	-25.9±0.2	-26.9±0.2	-25.7±0.5	-27.0±0.2	-27.2±0.3
2.68	1,2-Butadiene, 3-methoxy-	PS	-25.4±0.0	-25.8±0.1	-25.6±0.3	-23.8±0.4	-26.3±0.2	-23.2±0.3	-26.6±0.3	-26.5±0.2
2.92	Furfural	PS	-25.0±0.1	-25.4±0.1	-25.3±0.3	-23.7±0.2	-25.7±0.2	-22.8±0.3	-26.4±0.2	-26.0±0.4
3.06	Butanoic acid, 3-methyl-	PS	-26.9±0.2	-26.7±0.1	-26.2±0.3	-25.8±0.4	-26.5±0.2	-24.2±0.2	-27.0±0.3	-26.9±0.2
3.53	2,3,5-Trimethylfuran	PS	-26.8±0.1	-27.3±0.1	-26.6±0.2	-26.8±0.0	-26.8±0.3	-24.3±0.3	-27.6±0.3	-27.4±0.3
3.62	1,2-Cyclopentanedione	PS	-25.8±0.0	-26.0±0.1	-25.6±0.3	-25.9±0.0	-25.9±0.3	-23.5±0.3	-26.7±0.2	-26.5±0.3
3.96	5-Methylfurfural	PS	-26.1±0.1	-26.5±0.0	-26.2±0.2	-26.6±0.0	-26.5±0.3	-23.7±0.3	-27.0±0.3	-26.7±0.3
4.12	Phenol	ARO	-27.2±0.1	-27.7±0.1	-27.4±0.3	-27.5±0.3	-27.5±0.3	-24.7±0.3	-27.7±0.3	-27.7±0.2
4.30	3-Methyl hydantoin	N	-27.0±0.1	-26.9±0.2	-26.4±0.1	-26.9±0.5	-27.2±0.3	-23.7±0.3	-27.0±0.3	-26.8±0.2
4.60	2-Cyclopenten-1-one, 2-hydroxy-3-methyl-	PS	-25.7±0.1	-26.4±0.1	-25.8±0.2	-26.2±0.1	-26.2±0.1	-22.8±0.3	-26.4±0.1	-26.6±0.3
4.79	Benzeneacetaldehyde	ARO	-26.4±0.1	-26.9±0.1	-26.2±0.3	-27.0±0.1	-26.4±0.2	-23.5±0.3	-26.6±0.3	-26.6±0.2
5.10	Phenol, 3-methyl-	ARO	-26.8±0.2	-27.4±0.1	-27.0±0.4	-27.9±0.0	-26.5±0.2	-23.4±0.4	-26.9±0.1	-27.8±0.2
5.28	Guaiacol	LG	-27.9±0.2	-28.2±0.2	-28.3±0.3	-28.2±0.2	-27.8±0.3	-25.3±0.2	-28.7±0.1	-28.1±0.2
5.56	Levoglucosenone	PS	-27.0±0.2	-26.9±0.2	-26.2±0.2	-26.6±1.2	-26.8±0.2	-23.9±0.3	-28.1±0.3	-27.0±0.1
6.46	Methyl guaiacol	LG	-27.2±0.2	-27.8±0.2	-26.9±0.3	-25.6±0.3	-27.2±0.1	-24.2±0.5	-27.2±0.3	-27.6±0.3
6.79	4-Vinylphenol	LH	-28.9±0.0	-28.1±0.1	-27.0±0.4	-26.5±0.3	-27.6±0.2	-24.8±0.4	-27.2±0.0	-27.5±0.0
7.78	Indole	N	-28.2±0.5	-27.9±0.3	-27.9±0.4	-22.5±2.2	-27.3±0.2	-24.9±0.3	-28.5±0.1	-27.4±0.3
7.99	4-Vinylguaiacol	LG	-34.9±1.0	-29.5±0.7	-28.7±0.3	-34.4±2.7	-28.2±0.1	-27.6±0.5	-27.7±0.2	-27.9±0.1
8.06	Imidazole, 2-acetamino-5-methyl-	N	-26.3±0.8	-28.2±0.4	-27.5±0.2	-29.1±0.1	-27.7±0.2	-20.0±0.8	-27.4±0.4	-26.7±0.6
8.43	Syringol	LS	-26.5±2.0	-28.7±0.6	-28.2±0.3	-30.4±0.3	-27.7±0.3	-25.2±0.5	-27.7±0.3	-26.2±1.4
9.11	Vanillin	LG	-29.4±1.2	-29.6±0.5	-27.9±0.3	-29.2±0.4	-27.1±1.0	-24.8±0.4	-28.1±0.7	-23.4±1.1
9.67	Isoeugenol	LG	-26.3±2.1	-29.7±0.5	-28.1±0.3	-39.8±0.3	-28.2±0.1	-24.3±0.2	-31.7±1.5	-25.1±0.1
10.06	Benzene, 3-ethyl-1,2,4,5-tetramethyl-	ARO	-33.6±0.9	-30.4±1.3	-29.4±1.2	-34.5±0.4	-28.6±0.1	-26.5±0.1	-28.7±0.2	-31.6±0.8
10.15	Acetovanillone	LG	-28.6±0.1	-38.3±1.1	-29.0±0.2	-28.0±1.6	-29.5±0.1	-29.5±0.3	-33.1±0.6	-26.8±1.8
10.49	Levoglucosan + LG Methyl vanillate	PS	-16.5±0.3	-20.6±0.5	-23.6±1.2	-23.5±0.0	-23.9±0.6	-20.8±0.5	-22.7±0.4	-23.3±0.1
12.38	4-Propynylsyringol	LS	-34.0±0.3	-30.7±1.1	-37.1±0.0	-29.4±0.6	-32.2±0.3	-28.5±0.3	-28.6±0.1	-33.7±1.5
12.58	Methoxyeugenol	LS	-36.3±0.5	-35.1±0.8	-27.9±2.3	-28.8±0.8	-25.1±0.0	-27.1±0.5	-30.7±0.3	-24.4±0.1
12.78	4-Acetylsyringol	LS	-24.9±0.6	-24.9±0.4	-24.3±0.2	-24.1±0.3	-29.5±0.2	-21.1±0.3	-24.9±0.2	-27.6±0.0
13.13	Tetradecanoic acid (Fa_{14})	FA	-30.7±1.7	-30.9±2.7	-32.4±2.4	-29.3±0.5	-31.8±0.3	-29.1±0.6	-29.3±0.5	-31.0±0.2

13.45	Alkane C_{18}	AL	-36.4±0.4	-36.3±0.2	-37.5±1.2	-34.9±1.0	-36.6±0.0	-30.1±0.4	-27.3±0.1	-33.7±1.6
13.79	Pentadecanoic acid (Fa_{15})	FA	-29.7±0.2	-21.3±2.6	-24.4±0.7	-26.7±0.5	-27.0±0.1	-19.3±1.1	-29.0±0.5	-19.4±0.7
14.46	Alkene $C_{19:1}$	AL	-26.3±0.1	-25.4±0.2	-27.7±0.0	-27.1±0.5	-32.6±1.1	-27.4±0.8	-27.1±0.2	-27.4±1.1
14.53	Alkane C_{19}	AL	-32.0±0.6	-34.0±0.5	-34.1±0.1	-31.1±0.4	-32.4±0.4	-28.1±0.5	-30.9±0.3	-35.5±1.1
14.80	Hexadecanoic acid, methyl ester ($FaMe_{16}$)	FAME	-30.0±0.2	-32.6±0.3	-30.2±0.3	-30.9±0.3	-37.3±0.2	-31.2±0.7	-36.7±0.2	-34.2±0.7
15.19	*n*-Hexadecanoic acid (Fa_{16})	FA	-29.9±0.0	-33.8±0.1	-35.1±0.0	-34.5±0.3	-30.8±0.1	-28.8±0.2	-31.9±0.4	-32.2±0.0
15.47	Alkene $C_{20:1}$	AL	-36.5±0.1	-32.2±0.3	-30.4±0.3	-29.2±0.6	-34.2±0.1	-33.0±0.2	-36.3±0.7	-35.1±0.1
15.52	Alkane C_{20}	AL	-34.6±0.7	-34.0±0.1	-32.2±0.9	-32.8±1.3	-38.3±1.6	-33.7±0.1	-35.0±0.1	-37.0±0.5
16.41	Alkene $C_{21:1}$	AL	-32.0±0.3	-32.1±0.3	-37.8±0.6	-30.3±0.4	-33.5±0.0	-31.3±0.6	-34.1±1.1	-35.1±0.2
16.46	Alkane C_{21}	AL	-33.0±0.4	-34.9±0.0	-34.0±1.0	-34.0±0.4	-33.7±0.3	-33.2±0.8	-33.0±0.7	-30.6±0.3
16.87	Octadecenoic acid (Oleic acid)	FA	-25.0±0.1	-34.2±0.8	-36.7±0.5	-34.7±0.2	-36.3±1.8	-33.5±1.0	-27.6±2.7	-26.7±0.7
17.04	Octadecanoic acid (Fa_{18})	FA	-28.4±0.4	-32.1±0.6	-40.0±0.1	-30.5±0.1	-29.7±0.4	-29.0±1.6	-31.4±0.2	-31.0±1.0
17.31	Alkene $C_{22:1}$	AL	-31.8±0.3	-31.0±0.5	-32.8±0.1	-33.8±0.3	-33.1±0.2	-30.5±0.3	-33.1±0.4	-32.9±0.2
17.35	Alkane C_{22}	AL	-28.2±0.0	-31.2±0.5	-30.5±0.5	-31.2±0.3	-34.3±0.0	-32.5±2.8	-30.9±0.6	-28.4±0.2
18.18	Alkene $C_{23:1}$	AL	-35.0±0.9	-30.1±0.2	-33.1±0.3	-35.6±1.0	-33.0±0.8	-31.0±0.7	-34.0±0.1	-34.6±0.9
18.21	Alkane C_{23}	AL	-35.3±0.2	-34.0±2.0	-34.1±0.1	-34.8±1.6	-34.3±0.0	-27.9±1.3	-34.2±0.7	-33.7±0.7
19.00	Alkene $C_{24:1}$	AL	-34.7±0.8	-35.3±0.5	-31.2±1.2	-30.7±2.5	-33.0±0.2	-32.5±0.6	-33.1±0.1	-33.4±1.3
19.06	Alkane C_{24}	AL	-36.6±0.4	-35.1±0.6	-34.9±0.2	-35.4±0.0	-34.3±0.2	-30.6±0.0	-33.4±0.1	-34.8±0.2
19.96	Alkene $C_{25:1}$	AL	-38.6±0.7	-37.0±0.3	-35.7±0.8	-36.3±0.1	-34.1±0.3	-32.8±0.5	-33.7±0.9	-34.8±0.3
20.01	Alkane C_{25}	AL	-40.6±1.2	-39.5±0.8	-40.8±0.2	-38.4±0.7	-34.5±0.1	-34.7±0.5	-32.9±0.6	-37.1±0.6
20.72	Alkene $C_{26:1}$	AL	-36.0±0.5	-32.9±0.9	-37.5±0.4	-35.0±0.1	-36.1±0.1	-33.9±0.3	-34.8±0.2	-35.3±0.1
20.79	Alkane C_{26}	AL	-39.0±0.8	-37.5±0.9	-41.5±0.7	-41.1±1.3	-36.6±0.7	-34.2±0.4	-35.2±0.3	-37.4±0.2
21.34	Alkane C_{27}	AL	-38.8±0.8	-36.4±0.1	-38.4±0.6	-35.3±1.8	-38.8±0.9	-34.5±0.3	-34.7±0.2	-34.5±0.1
21.57	Tetracosanoic acid, methyl ester ($FaMe_{24}$)	FAME	-32.2±1.8	-34.9±0.9	-34.8±0.1	-33.4±0.5	-32.5±0.7	-30.1±0.3	-31.7±0.1	-32.6±0.1
21.88	15-Tetracosenoic acid, methyl ester ($FaMe_{24}$)	FAME	-33.4±1.1	-34.6±1.0	-38.4±0.5	-34.8±0.5	-36.1±1.7	-34.1±0.8	-33.5±0.0	-32.7±1.2
22.03	Alkane C_{28}	AL	-39.6±0.3	-37.0±0.6	-36.1±0.7	-37.3±0.5	-41.5±0.5	-35.6±1.2	-35.8±0.2	-37.1±0.2
22.74	Alkane C_{29}	AL	-42.0±0.5	-36.2±0.5	-36.1±0.3	-35.9±1.2	-37.0±0.3	-34.4±0.3	-36.0±0.4	-35.1±0.0
23.47	Alkane C_{30}	AL	-39.5±0.3	-31.9±0.3	-38.8±0.9	-28.0±0.1	-36.2±0.1	-35.3±0.1	-37.9±0.2	-38.3±0.4
24.32	Alkane C_{31}	AL	-43.2±2.0	-37.7±0.4	-36.3±0.3	-35.8±2.3	-36.9±0.1	-34.1±0.1	-37.5±0.7	-39.0±0.2

Table S2. List of pyrolysis products, with their retention times (RT, in minutes), biogenic compound (origin) and their δ^{2}H values in each of the climatic treatment within each habitat (average expressed as ‰; *n* = 3 ± standard deviation).

			**Open**				**Tree**			
**RT**	**Compound**	**Origin**	**C**	**W**	**D**	**W+D**	**C**	**W**	**D**	**W+D**
2.45	Pyridine	N	-111.1±4.8	-110.1±2.4	-111.2±1.2	-80.9±1.7	-74.6±1.5	-81.2±0.8	-78.3±2.1	-61.1±2.7
2.68	1,2-Butadiene, 3-methoxy-	PS	-102.8±1.4	-94.3±3.2	-98.5±2.1	-51.3±3.9	-49.0±1.6	-49.9±3.3	-42.2±0.6	-38.6±2.0
2.92	Furfural	PS	-78.3±4.9	-86.5±3.8	-72.5±3.4	-40.8±2.0	-29.4±2.1	-28.7±0.8	-20.8±1.7	-13.9±2.4
3.06	Butanoic acid, 3-methyl-	PS	-79.8±7.4	-83.2±5.7	-86.5±1.6	-45.3±2.7	-48.7±2.0	-51.5±2.1	-44.6±2.4	-28.4±0.8
3.53	2,3,5-Trimethylfuran	PS	-96.2±0.1	-94.1±3.8	-97.7±5.1	-53.0±4.4	-33.9±1.0	-45.2±1.2	-29.5±1.0	-33.2±1.8
3.62	1,2-Cyclopentanedione	PS	-91.2±3.9	-91.9±4.3	-92.9±2.6	-51.8±4.4	-43.0±1.1	-51.6±2.5	-37.6±1.4	-31.5±1.9
3.96	5-Methylfurfural	PS	-96.4±2.0	-92.7±4.3	-91.2±0.5	-47.1±1.8	-40.3±1.6	-44.9±2.6	-32.5±2.2	-26.5±2.1
4.12	Phenol	ARO	-101.0±0.7	-101.3±0.7	-97.6±2.4	-55.9±1.4	-51.1±1.3	-56.3±2.3	-43.6±1.3	-38.5±1.6
4.30	3-Methyl Hydantoin	N	-93.0±0.5	-96.9±2.7	-95.9±0.8	-50.6±2.2	-49.9±1.8	-54.4±2.2	-45.2±2.4	-34.4±1.3
4.60	2-Cyclopenten-1-one, 2-hydroxy-3-methyl-	PS	-100.5±3.5	-95.6±1.6	-92.2±1.2	-48.8±2.8	-45.9±2.0	-51.3±2.4	-42.6±2.8	-31.6±1.6
4.79	Benzeneacetaldehyde	ARO	-97.5±1.4	-102.5±3.5	-96.4±0.9	-50.9±1.6	-50.6±1.3	-56.2±2.2	-45.7±2.7	-36.1±1.2
5.10	Phenol, 3-methyl-	ARO	-106.0±8.0	-102.6±1.2	-100.8±2.2	-56.6±3.0	-53.9±1.0	-58.7±2.0	-46.1±1.5	-41.3±0.9
5.28	Guaiacol	LG	-96.4±1.5	-100.6±1.2	-97.9±3.4	-49.7±2.4	-49.1±0.7	-55.6±2.2	-41.3±0.4	-34.9±2.6
5.56	Levoglucosenone	PS	-123.6±2.8	-103.5±5.6	-91.2±1.7	-48.8±4.5	-44.1±1.0	-50.4±3.3	-40.5±2.9	-33.1±2.0
6.46	Creosol (Methyl guaiacol)	LG	-80.3±0.6	-88.0±0.8	-64.3±1.0	-42.3±2.1	-30.7±0.3	-32.1±3.0	-21.2±1.8	-19.0±0.1
6.79	4-vinylphenol	LH	-76.8±1.1	-93.9±2.9	-73.0±2.0	-41.5±7.7	-35.9±1.9	-41.2±2.0	-29.6±2.3	-21.6±1.3
7.99	Indole	N	-94.6±5.6	-98.1±0.6	-78.5±4.0	-42.8±0.6	-48.8±0.8	-55.6±0.9	-42.5±2.0	-33.0±1.1
8.06	4-Vinylguaiacol	LG	-92.8±6.1	-102.3±0.6	-100.0±3.8	-66.2±1.8	-53.1±1.1	-64.2±5.8	-46.4±2.7	-36.3±0.3
8.43	Imidazole, 2-acetamino-5-methyl-	N	-59.6±0.6	-98.5±0.4	-87.3±7.2	-42.2±5.4	-47.0±1.9	-85.8±11.1	-43.4±1.9	-30.6±0.7
9.11	Syringol	LS	-67.0±3.2	-100.0±0.7	-121.7±4.7	-122.4±0.8	-54.5±1.3	-86.3±0.2	-51.0±2.6	-39.3±1.3
9.67	Vanillin	LG	-58.0±6.0	-96.5±1.6	-72.4±2.7	-60.8±4.1	-41.8±1.4	-51.3±1.3	-86.0±2.4	-30.8±1.2
10.06	Isoeugenol	LG	-115.6±10.2	-96.1±0.6	-86.0±1.2	-151.5±7.3	-52.4±0.1	-103.0±0.8	-64.1±0.9	-56.0±0.6
10.15	Benzene, 3-ethyl-1,2,4,5-tetramethyl-	ARO	-96.1±4.6	-99.3±1.0	-71.8±1.6	-32.2±0.6	-38.0±3.8	-59.7±6.8	-51.4±0.2	-32.0±3.7
10.49	Acetovanillone	LG	-109.7±8.2	-98.9±0.3	-61.3±0.1	-38.9±2.0	-56.2±2.0	-56.3±3.3	-36.5±6.5	-87.3±4.8
12.58	Propynylsyringol	LS	-114.5±11.5	-111.6±5.6	-167.8±4.6	-130.0±8.9	-221.7±5.7	-207.5±3.4	-178.6±11.4	-184.8±3.3
12.78	Methoxyeugenol	LS	-78.5±2.5	-102.8±0.4	-80.5±6.8	-75.0±0.4	-48.1±0.1	-26.3±2.5	-35.0±2.9	-29.1±1.8
13.13	4-Acetylsyringol	LS	-92.9±14.6	-92.4±4.5	-108.7±2.2	-97.3±7.9	-73.8±0.2	-41.1±3.2	-80.2±7.7	-74.0±3.8
13.45	*n*-Tetradecanoic acid (Fa_{14})	FA	-161.9±1.8	-112.7±9.6	-117.4±0.7	-59.0±3.5	-91.4±1.5	-79.1±8.8	-91.2±1.4	-96.9±1.8
14.46	*n*-Pentadecanoic acid (Fa_{15})	FA	-58.2±3.7	-99.4±4.0	-95.9±2.5	-103.7±8.5	-123.2±1.3	-116.2±0.3	-130.5±10.8	-65.3±1.8
14.80	Alkane C_{19}	AL	-73.0±1.9	-105.5±5.0	-102.0±10.7	-62.2±8.3	-101.0±0.3	-57.9±0.6	-53.8±4.4	-50.4±1.1
15.47	Hexadecanoic acid, methyl ester ($FaMe_{16}$)	FAME	-121.6±0.8	-124.0±0.4	-124.0±0.3	-101.1±3.6	-124.9±5.2	-123.3±3.0	-118.0±3.5	-80.3±10.8
15.52	*n*-Hexadecanoic acid (Fa_{16})	FA	-78.7±2.7	-88.8±4.4	-104.7±5.9	-32.0±2.9	-61.0±2.0	-67.0±1.5	-61.3±2.7	-49.4±1.8
16.46	Alkane C_{20}	AL	-105.3±5.0	-89.6±4.2	-54.6±5.1	-31.0±5.1	-69.8±3.9	-79.7±4.9	-48.0±4.5	-31.3±7.1
17.31	Alkane C_{21}	AL	-100.0±1.7	-120.2±3.1	-60.3±3.7	-54.7±1.4	-53.7±3.6	-38.7±2.9	-37.7±3.2	-29.4±3.4
17.35	Octadecenoic acid (Oleic acid)	FA	-41.2±3.7	-80.4±4.9	-90.0±0.8	-109.0±3.2	-71.8±5.1	-55.2±1.8	-48.9±6.6	-52.8±9.5

18.18	*n*-Octadecanoic acid (Fa_{18})	FA	-68.5±0.3	-65.9±10.9	-99.8±1.8	-113.4±6.5	-67.9±3.9	-63.8±4.6	-53.5±3.6	-46.6±1.2
19.00	Alkane C_{22}	AL	-120.7±1.5	-96.1±6.2	-125.7±1.6	-102.5±9.6	-147.2±1.4	-91.7±5.2	-87.4±0.8	-83.0±11.0
20.01	Alkane C_{23}	AL	-122.2±11.7	-143.3±5.5	-167.8±5.1	-148.0±3.8	-137.0±1.0	-117.6±2.0	-91.1±6.7	-133.1±0.8
21.57	Alkane C_{24}	AL	-127.9±5.8	-107.6±1.8	-125.3±8.4	-69.5±5.1	-87.9±2.0	-69.9±7.6	-91.9±2.0	-80.3±1.1
22.74	Alkane C_{25}	AL	-143.4±2.9	-107.5±2.2	-107.4±1.0	-37.5±2.5	-83.2±1.4	-93.5±3.6	-75.8±2.9	-62.5±2.0
20.79	Alkane C_{26}	AL	-150.1±8.0	-117.7±3.9	-92.5±1.9	-37.3±1.2	-83.8±0.9	-82.9±1.9	-37.1±0.4	-54.2±4.7
21.34	Alkane C27	AL	-136.2±8.0	-126.6±9.5	-82.7±4.7	-103.9±12.7	-92.7±4.5	-65.8±1.2	-68.1±5.1	-57.1±1.0
21.57	Tetracosanoic acid, methyl ester ($FaMe_{24}$)	FAME	-99.3±9.1	-114.5±1.3	-97.3±2.2	-63.6±2.3	-115.3±1.3	-128.0±3.7	-69.5±0.8	-104.5±5.0
21.88	15-Tetracosenoic acid, methyl ester, (Z)- ($FaMe_{24}$)	FAME	-67.9±8.7	-125.9±7.2	-136.7±4.6	-79.0±4.2	-54.3±0.3	-106.7±1.7	-78.1±9.6	-92.7±3.1
22.03	Alkane C_{28}	AL	-112.6±2.1	-74.5±10.0	-68.1±10.7	-79.6±0.8	-86.4±1.5	-61.9±0.0[b]	-77.3±4.0	-76.1±0.2
22.74	Alkane C_{29}	AL	-113.7±0.2	-77.9±7.9	-76.4±3.2	-98.7±2.5	-87.4±6.9	-79.2±1.9	-76.6±2.4	-62.9±4.7
23.47	Alkane C_{30}	AL	-145.8±3.6	-114.6±2.0	-106.4±0.5	-63.5±4.4	-127.9±2.1	-103.9±10.8	-131.9±0.1	-67.6±3.6
24.32	Alkane C_{31}	AL	-168.4±5.5	-92.4±1.9	-129.3±0.9	-81.7±1.5	-65.9±3.0	-75.7±1.1	-66.3±2.0	-52.5±1.7
